# The impact of liver transplantation on endpoint selection in alcohol-associated hepatitis trials

**DOI:** 10.1097/HC9.0000000000000709

**Published:** 2025-04-30

**Authors:** Suthat Liangpunsakul, William B. Krebs, Allison J. Kwong, Paul Y. Kwo, Robert S. Brown, WeiQi Lin, Norman L. Sussman

**Affiliations:** 1 Department of Medicine, Division of Gastroenterology and Hepatology, Indiana University School of Medicine, Indianapolis, Indiana, USA; 2 Krebs Consulting Statistician, Santa Rosa, CA, USA﻿; 3 Department of Medicine, Division of Gastroenterology and Hepatology, Stanford University School of Medicine, Palo Alto, California, USA; 4 Department of Medicine, Division of Gastroenterology and Hepatology, Weill Cornell Medicine, New York, New York, USA; 5 Durect Corporation, Cupertino, California, USA; 6 Division of Gastroenterology and Hepatology, Department of Medicine, ﻿Baylor College of Medicine, Houston, Texas, USA

**Keywords:** alcohol-associated hepatitis, clinical trials, statistical methods

## Abstract

**Background:**

Alcohol-associated hepatitis (AH) is a serious liver disease caused by heavy alcohol consumption with severe cases exhibiting a 90-day mortality rate of ~30%. No drugs have been approved for AH, and regulatory approval currently requires evidence of improved survival. The lack of effective drug therapies and high mortality rates have fueled interest in early liver transplantation (LT), which has a survival rate that exceeds 90%. However, LT is resource-intensive and is available only in expert centers, where most AH trials are conducted. As a result, LT is overrepresented in recent AH studies, leading to confounding and unresolved questions regarding valid endpoints in therapeutic AH trials.

**Methods:**

We propose methodological approaches to address the inclusion of LT in AH trials, supported by power calculations and data from the AHFIRM trial, a 300-patient multicenter study completed in late 2023. We demonstrate the impact of effect size, trial size, and statistical methods on trial design and interpretation.

**Results:**

Effect size plays a crucial role in power calculations. While 90-day survival is the most efficient endpoint, competing risk analysis, primary stratum analysis, and win ratio are valuable tests for assessing the role of LT. The combined endpoint of death or LT is the least efficient method and requires the largest trial population to achieve statistical significance. We recommend using multiple statistical methods with adjustments for multiplicity.

**Conclusions:**

The adoption of early LT complicates the assessment of new therapies for AH. Statistical methods and endpoints are critical in power calculations and when assessing the efficacy of new therapeutic agents. We recommend mortality as the primary analysis complemented by hierarchical secondary analyses that avoid problems of multiplicity.

## Graphical abstract

**Figure:**
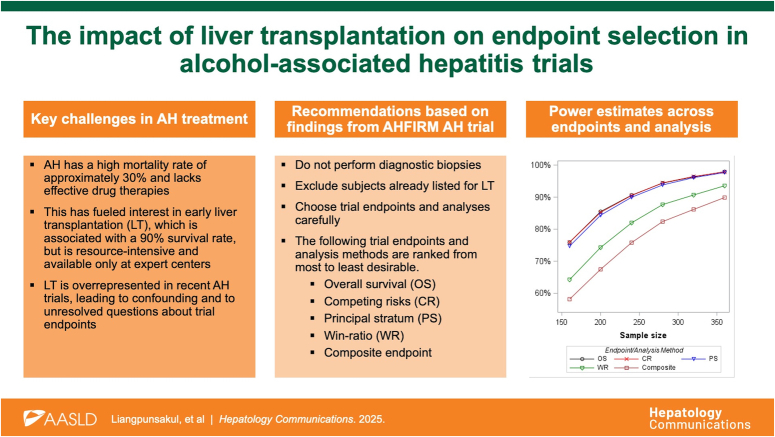

## INTRODUCTION

Alcohol-associated hepatitis (AH) is a severe inflammatory liver condition caused by heavy alcohol consumption. It is characterized histologically by hepatic steatosis, ballooning degeneration, neutrophilic infiltration, megamitochondria, Mallory hyaline, and variable fibrosis.^1^ A clinical diagnosis of probable AH (ie, without a compatible liver biopsy) is widely accepted in clinical trials.^2^ Severe AH, typically defined as a MELD score >20 and/or a Maddrey Discriminant Function ≥32, has a reported 90-day mortality rate of ~30%.^3^^–^^5^ For decades, efforts to develop effective medical therapies for AH have been unsuccessful, leading to frustration and diminished research interest. However, the COVID-19 pandemic renewed focus on AH due to increased alcohol consumption with a consequent surge in alcohol-associated hospital admissions, deaths, and liver transplants.^6^^–^^10^

Liver transplantation (LT) was historically unavailable to patients with AH until they had demonstrated 6 months of sobriety, a policy lacking a scientific basis and resulting in preventable deaths before patients could meet the requirement.^11^ A pivotal study that demonstrated a strong survival benefit of LT for patients with AH^12^ led the American Association for the Study of Liver Diseases (AASLD) and the European Society for the Study of the Liver (EASL) to recommend individualized approaches over rigid time-based sobriety criteria.^13^ Since then, early LT for AH has gained wide acceptance in several countries, including the United States.^8^^,^^14^^,^^15^

This LT policy shift benefits some patients with AH but creates challenges for researchers and companies developing new therapies. One key issue is that most liver experts work in transplant centers where AH trial participants, especially those with severe AH, may also qualify for LT. As a result, studies conducted in these centers are influenced by LT outcomes that are not available to the vast majority of patients with AH. A challenge arises once a patient undergoes LT because it is impossible to determine whether recovery and spontaneous survival are possible without the transplant. LT may obscure the ineffectiveness of therapy by rescuing a patient who might otherwise have died. Conversely, an effective therapy could stabilize a patient, allowing more time to receive a transplant. These opposing interpretations have led to a lack of consensus within the hepatology community on how to evaluate this postrandomization event.^16^

## METHODS

In this paper, we propose statistical approaches to address the challenges posed by LT in AH trials that aim for regulatory drug approval. We provide power and sample size calculations based on data from the AHFIRM Trial (NCT04563026),^17^ illustrating a range of sample sizes and effect sizes. The key inclusion and exclusion criteria for the AHFIRM trial are shown in Table 1. This manuscript focuses on outcome endpoints since surrogate markers are both imprecise and insufficient for regulatory approval.

**TABLE 1:** Key inclusion and exclusion criteria for AHFIRM

Inclusion criteria	Exclusion criteria
• Clinical diagnosis of AH^2^ • Maddrey discriminant function >32 • MELD score 21–30 • Agreement to participate in an alcohol abstinence program determined by the study center	• Pretreatment systemic steroids >8 d • High risk of alcohol withdrawal seizures or delirium tremens • Active infection including hepatitis, HIV, and sepsis • Serum creatinine >2.5 mg/dL or CVVH • Uncontrolled gastrointestinal bleeding • Grade 3 or higher HE • Preceding refractory ascites • An incompatible liver biopsy (if performed)

### Patient selection and randomization

Entry criteria for AH trials have been simplified with the adoption of a widely accepted clinical diagnosis and of the MELD score as a validated severity scale.^2^ While some experts advocate for liver biopsy in AH studies, the diagnosis of AH cannot be made on histology alone, and its value as a diagnostic or prognostic tool remains inconclusive. Misdiagnosed AH may not qualitatively affect a trial if errors are balanced across treatment groups, but enrolling too many patients with other diagnoses could obscure the effects of a therapeutic agent. As reported by Verma and colleagues, clinical criteria demonstrated strong predictive value in severe AH. Studies using a MELD score cutoff for severe AH of >20 showed an accuracy of 91.6% with 95% CIs (87.3%, 94.6%); and studies using a serum bilirubin cutoff of 5 mg/dL instead of 3 mg/dL showed an accuracy of 88.5% with 95% CI (85.2%, 91.1%).^18^ These findings support the use of clinical criteria in severe AH trials. In fact, liver biopsies may hinder AH studies for several reasons. First, confirmatory biopsies are not routinely performed in many liver centers. The AHFIRM trial, which enrolled patients across the United States, Australia, France, Belgium, and the United Kingdom, allowed investigators to choose biopsy confirmation, yet only 25 of 307 patients underwent biopsy (5/232 in the United States, 3/41 in Australia, 17/26 in France and Belgium, and 0/8 in the United Kingdom). Some experts view confirmatory biopsies as unnecessary or not in the patient’s best interest since they have not been shown to improve AH management and carry risks of severe complications. Reluctance to perform biopsies may hinder enrollment, jeopardizing trial completion. Second, the time required for biopsy and pathology review may delay treatment initiation, potentially disqualifying patients. For instance, an anticipated phase III trial of larsucosterol limits the time from hospital admission to dosing to 9 days.^17^ Third, requiring liver biopsy could restrict drug labeling or limit access to a potentially beneficial therapy in patients lacking biopsy confirmation, confining treatment to specialized centers. Lastly, biopsy-based trials may inadvertently select a subset of patients rather than representing the broader AH population.

The randomization protocol in any study should include matching patient populations for disease characteristics, disease severity, and patient demographics. For example, abbreviated entry and exclusion criteria for the AHFIRM trial are shown in Table 1. An additional concern in AH trials is the center-to-center variability in “standard of care,” specifically regarding differences in preference for corticosteroids and early versus delayed LT. These postrandomization differences can be addressed by matching practices across sites but are most effectively mitigated by site-level randomization, ensuring that practices are applied equally to both treated and control patients at each site.

### Endpoint timing

The short-term mortality rate in severe AH is alarmingly high, with ~30% of patients dying within 90 days.^3^^–^^5^^,^^19^ This underscores the need to assess survival outcomes directly in patients with severe AH. However, determining the optimal timepoint for survival or mortality assessment has been debated. Mortality at 28 days, although commonly used in earlier studies, fails to capture the full trajectory of AH because it excludes ongoing disease progression and the risk of mortality beyond this timeframe.^20^ Conversely, mortality rates at 6 or 12 months are often influenced by factors unrelated to progressive AH, such as a return to harmful alcohol use, which complicate the interpretation of outcomes at these later intervals.^20^^,^^21^ Recent reviews and consensus documents recommend a 90-day timepoint for outcome measurement.^16^^,^^22^^,^^23^ Standardizing this endpoint enhances comparability across trials and supports future meta-analyses, ultimately improving patient care and advancing drug development in AH.

### The liver transplant conundrum

LT complicates the interpretation of efficacy in clinical studies of AH therapies. In such studies, LT is an *intercurrent event*, an event that occurs between patient randomization and final assessment that is not determined by the randomization procedure, and which impacts the outcome of interest. LT cannot be predicted at enrollment, and it raises 90-day survival rates from 70% to over 90%,^24^ but the decision to transplant may depend on factors beyond the patient’s immediate medical condition. For instance, a transplant committee might opt for LT, not because of a risk of death within 90 days, but because of concerns about long-term outcomes. Specifically, the committee may act preemptively to avoid chronic liver insufficiency (commonly referred to as “MELD purgatory“^25^) if the patient fails to re-compensate.^26^ Although LT is a definitive rescue therapy, not every transplant rescues a patient from an otherwise fatal outcome; a patient who is not selected for transplant may very well survive through the trial’s defined period. As noted, there is no reliable method to determine whether a patient with AH who undergoes LT would have died without it, and transplant teams must make decisions based on the best available information at the time of listing and transplantation. In addition, geographic variation in donor availability and center practices may influence whether a patient with severe AH undergoes LT. The most effective way to balance transplant policies in AH trials is to randomize patients at each site. Some have suggested conducting AH trials at centers that do not offer LT, which may seem like an obvious solution. However, this strategy is flawed because nontransplant centers frequently lack the patient population and resources necessary to conduct complex inpatient studies. Moreover, nontransplant centers generally have relationships with transplant centers and are able to transfer patients when necessary, thus complicating follow-up and increasing the likelihood of missing data. Given the current state of LT in the United States, studies will be conducted on a population that resembles but is not identical to the broader AH population. Possible methods for analyzing data in AH clinical trials in the context of LT are outlined in Table 2.

**TABLE 2:** Statistical options for assessing outcome in AH trials

Method	Concept	Comment
Overall survival	LT occurs randomly in both groups and can be ignored	Pro: This is the most efficient method Con: A patient who is failing therapy may survive if “rescued” by LT
Competing risk	LT is a competing risk with death	Pro: Efficiency is equal to overall survival Con: LT may be done for reasons other than concern about death in the defined period (ie, not a true competing risk)
Principal stratum	Survival adjusted for LT as an intercurrent event	Pro: Efficiency is nearly equal to overall mortality Con: Omits some data from analysis
Win ratio	Ordered analysis of death and LT Note: If the ratio of death to LT is similar for all treatments, then the win ratio method is equivalent to the Composite Endpoint	Pro: Stratifies LT as a better outcome than death Con: Concern about the relative values of LT versus survival rates
Composite endpoint	Death and LT are equivalent	Pro: No major advantage Con: The least efficient method of analysis—may obscure a positive therapeutic effect

Abbreviations: AH, alcohol-associated hepatitis; LT, liver transplantation.

### Multiple comparisons and multiplicity

Given that LT can be interpreted as either a positive or negative outcome, clinical trials in AH should incorporate multiple analyses of endpoints. This raises the issue of multiplicity, a statistical challenge that occurs when multiple tests are conducted on the same data set, thus increasing the risk of false-positive findings. One approach to address this challenge is hierarchical testing, in which analyses are performed in a predefined sequence. In this framework, each subsequent analysis is conducted only if the preceding one achieves statistical significance. A proposed hierarchy for AH trials is detailed in Table 2. Another method involves adjusting the significance thresholds for hypothesis tests to account for multiple comparisons. This ensures that the overall false-positive rate remains controlled and aligns with the guidance outlined in the FDA’s document, *Multiple Endpoints in Clinical Trials: Guidance for Industry*.^27^ Both strategies offer effective ways to manage multiplicity, ensuring that trial results remain both reliable and meaningful for clinical interpretation.

## RESULTS

### Overall survival (accounting for the effect of LT)

Survival, or its complement, mortality, is a logical endpoint in a disease with a high risk of death over a relatively short period. Earlier studies of AH were unencumbered by the possibility of LT, but contemporary clinical trials, particularly those in the United States, are often conducted in institutions where LT is disproportionately represented. In overall survival analyses, the effect of LT is ignored under the assumption that patients in all study arms have an equal likelihood of receiving a transplant, especially if site-level randomization is used. This assumption could be challenged by a drug that rapidly and convincingly improves liver function.^28^ However, if, as suggested by the AHFIRM trial, LT rates are independent of treatment and mortality, an effective therapy might increase the number of transplants by prolonging the survival of patients listed for LT, thus allowing more time for a donor liver to become available.^16^^,^^17^ Studies, including results from the AHFIRM trial, show that patients with AH who are listed for LT often have high transplantation rates.^6^^,^^29^ Furthermore, the decision to proceed with LT, especially in the United States, is influenced by both clinical and psychosocial factors.^30^ Sponsors and investigators should, therefore, mitigate the impact of LT by balancing site and regional LT policies or using site-level randomization.

The primary challenge with an overall survival analysis is that LT may “rescue” patients who are failing treatment, leading them to be counted as survivors. This scenario risks making an inferior treatment appear superior if a significant proportion of patients are saved by LT. Recent studies, including AHFIRM, suggest this is not a major concern because randomization in clinical trials typically balances disease severity across treatment arms, and the decision to transplant often hinges on psychosocial factors rather than purely on clinical deterioration.^30^ Thus, LT may be viewed as an event balanced between the randomized study groups. Survival, regardless of LT, remains the simplest and most persuasive endpoint, as demonstrated in the AHFIRM trial and further illustrated below. This endpoint ensures clarity and comparability, making it our recommended cornerstone of AH clinical trial outcomes.

### Survival to day 90 adjusted for LT as a competing risk

**FIGURE 1:**
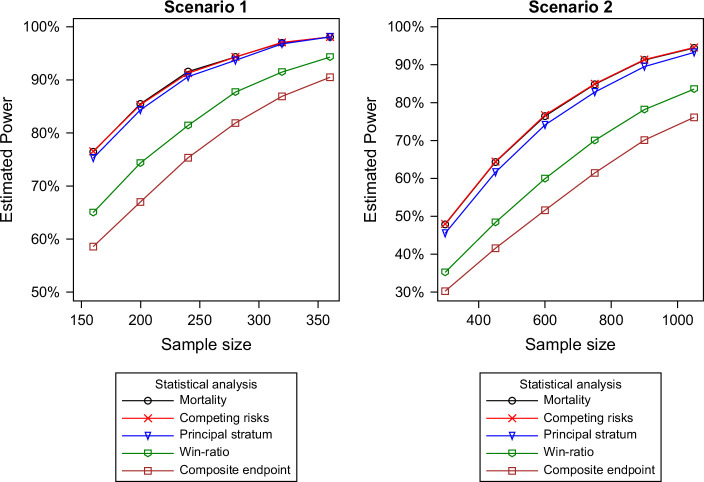
Simulated power estimates for 5 statistical methods. The figure illustrates the comparative efficiency of the 5 methods, showing that they perform equally well in both scenarios. The mortality and competing risk methods are essentially equivalent and overlap in the figures. The principal stratum method is nearly as efficient as the first 2 though it sacrifices some efficiency by excluding patients who undergo LT from the analysis. The win ratio analysis is less efficient than the first 3 methods, with its power diminished by the significant overlap between the experimental and control distributions. Lastly, the composite endpoint is less efficient than the win ratio, as it does not account for the distinction between deaths and transplants, which is incorporated in the win ratio method. Abbreviation: LT, liver transplantation.

The confounding effect of LT on mortality assessment necessitates a nuanced analytical approach. In time-to-event analyses, treating LT as a competing risk allows for an unbiased evaluation of the survival outcome of interest. A common method is to compare the cumulative incidence of death between treatment groups, which can be visually represented using cumulative incidence curves. These curves can be statistically compared using the Gray test.^31^ More advanced statistical models can incorporate covariates, including time-dependent ones, to further refine the analysis. For instance, the Fine-Gray model can estimate subdistribution hazards, or cause-specific hazards can be developed for death and LT events.^32^ Power simulations, as shown in Figure 1, indicate that the efficiency of the competing risk model is comparable to that of the overall survival model. This suggests that competing risk models provide a robust alternative for analyzing survival outcomes in the context of LT, enabling a clearer understanding of treatment effects in AH clinical trials.

### Survival in the absence of LT, principal stratum analysis

The International Council for Harmonization of Technical Requirements for Pharmaceuticals for Human Use guideline^33^ defines events that occur after treatment initiation and that have the potential to affect the interpretation of the measurement of interest as *intercurrent events*. LT is such an event because it prevents us from assessing survival at day 90, and thus acts as a censoring event.^34^ Survival to day 90, adjusted for LT as an intercurrent event, can be analyzed using the same methods as survival to day 90 on the principal stratum, which consists of subjects who remain in the study and do not receive a liver transplant by day 90.

Formally, the principal stratum of interest is the subset of patients who would not undergo transplantation under any therapy in the trial. This analysis relies on unobservable characteristics of study patients at enrollment. However, as discussed in the section on overall survival, LTs are independent, or nearly independent, of the treatment arm. In this case, the intercurrent event of LT can be ignored, and it is sufficient to compare study treatments in the subset of patients who do not receive liver transplants. It is important to note that, in this setting, both the number of deaths and the number of subjects observed are random variables. Therefore, statistical tests and estimators must account for both sources of random variation. As shown in the “Illustrations” section below, the principal stratum analysis is nearly as efficient as the overall mortality analysis.

### Analysis of transplant-free survival at day 90 by win-ratio

Possible outcomes at day 90 in an AH trial are to be alive and liver transplant–free, to be alive with a LT, or to die (death after LT in the United States is rare and may be excluded). These outcomes can be ordered based on the rule that being alive without LT is a better outcome than being alive after LT, and that both are better outcomes than death. To calculate the win ratio, each subject in the experimental group is compared to each subject in the placebo group. The proportions of cases where the experimental subject has a better outcome than the placebo subject, the placebo subject has a better outcome than the experimental subject, and both subjects have the same outcome are computed. The estimand of interest is the population difference between the proportion of subjects where the experimental agent yields a better outcome and the proportion where the placebo yields a better outcome.^35^ This population difference can be estimated by the corresponding sample difference.

The Wilcoxon midrank statistic can be used to test the null hypothesis that this difference is zero, and a large-sample CI for the difference should also be provided. The win ratio method is particularly suitable for situations where the treatments under study have notably different effects on death and LT. If the ratio of death to LT is substantially the same for all treatments, the win ratio method becomes equivalent to the composite endpoint method described below.

### Composite endpoint of death or liver transplant

This analysis equates LT with death under the assumption that every transplanted patient would have died by day 90 without the intervention of LT. As discussed, this claim cannot be made with certainty since it is not possible to definitively predict whether a patient will die or would have died by day 90 without LT. Predictive tools typically have an AUC of around 0.84.^4^^,^^19^ The composite endpoint method increases the incidence of events in both arms, which, in turn, raises the SE of the estimates for both the experimental and control groups. In our analysis of the AHFIRM trial, this method was the least efficient and required the largest number of patients when calculating power for future trials.

### Computational demonstration for 5 analysis methods

Analysis of a demonstration data set from the AHFIRM trial is shown in Tables 3, 4A, and 4B. Table 3 shows outcomes (deaths, LT, and survival without LT). Table 4A shows an analysis of deaths, and Table 4B shows an analysis by win probability with accompanying 95% CIs and *p* values. The results are from US subjects treated with larsucosterol 30 mg or placebo who had outcome data available (see Shiffman et al^17^ for a full report of the trial).

**TABLE 3:** Outcomes by treatment group: mortality, LT, and survival without LT for US data set (larsucosterol 30 mg vs. placebo)

Treatment	Died	LT	Alive w/o LT	Total
Active, n (%)	8 (11.0)	5 (6.9)	60 (82.2)	73
Control, n (%)	20 (26.0)	5 (6.5)	52 (67.5)	77

Abbreviation: LT, liver transplantation.

**TABLE 4A:** Analysis results for mortality for the US data set (larsucosterol 30 mg vs. placebo)

	Mortality estimate (std error)				
Analysis method	Active	Control	Difference	95% CI	*p*
Overall survival	0.11 (0.037)	0.26 (0.050)	−0.15 (0.062)	(−0.272, −0.029)	0.0183
Competing risks	0.11 (0.037)	0.26 (0.051)	−0.15 (0.063)	(−0.273, −0.027)	0.0175
Principal stratum	0.12 (0.039)	0.28 (0.053)	−0.16 (0.066)	(−0.289, −0.031)	0.0179
Composite endpoint	0.18 (0.045)	0.32 (0.053)	−0.15 (0.070)	(−0.283, −0.010)	0.0391

**TABLE 4B:** Analysis results for win probability for the US data set (larsucosterol 30 mg vs. placebo)

	Probability estimate (std error)				
Analysis method	Active better than control	Control better than active	Difference	95% CI	*p*
Win ratio	0.28 (0.043)	0.13 (0.031)	0.16 (0.069)	(0.022, 0.293)	0.0287

### Considerations for Corticosteroid use in clinical trials of AH

The therapeutic efficacy of corticosteroids in AH has been debated, with some studies indicating benefits in reducing short-term mortality, while others show minimal or no effect.^3^^,^^36^^,^^37^ Despite these mixed findings, multiple clinical guidelines recommend corticosteroids for eligible patients with severe AH.^2^^,^^38^^,^^39^ Allowing site investigators the discretion to use corticosteroids ensures individualized patient care, enabling clinical teams to provide what they consider the standard of care while still contributing to the broader understanding of AH treatment. In the AHFIRM trial, steroids were prescribed at the investigators’ discretion but were given only to patients in the placebo group—patients in the active (larsucosterol) arms received matching placebo capsules to avoid the confounding effects of steroids while maintaining the blind.

The use of steroids is also an intercurrent event, as previously described, and any inferences regarding their potential effect must be made cautiously. This is possible but requires identifying a group of patients in both the experimental and control groups who would be treated with steroids. Dividing the control group into an active steroid control arm and a steroid-free control arm may be valuable in early-phase clinical trials. However, a company focused on developing a therapeutic drug for AH may prefer to concentrate on whether a novel agent is more effective than the standard of care rather than delving into the specifics of steroid use in AH.

### Illustration of sample size estimation and power analysis for statistical methods in AH trials

To enhance the discussion of statistical methods above, we provide sample size estimates to highlight the comparison between different analysis methods. Two scenarios are presented, both derived from the recent AHFIRM trial in AH.^17^ Scenario 1 is based on the mortality and transplant data for US subjects in the larsucosterol 30 mg and placebo groups, while scenario 2 uses data from global subjects in the same groups. Sample size calculations were performed using SAS PROC POWER following standard methods. For the mortality, principal stratum, and composite endpoint methods, the sample sizes were calculated for studies comparing 2 frequencies. For the competing risk analysis, the sample size was computed for a study comparing 2 time-to-event variables with exponential distributions. The sample size for the win ratio method was computed for 2 discrete distributions using the Wilcoxon midrank sum statistic. Table 5 presents the analytical estimates of the sample sizes required to achieve 80% power for each of the 5e statistical methods across both scenarios. To provide additional context for the 5 statistical methods, a series of simulation runs were conducted to compare all methods using a common set of simulated data across a range of sample sizes, based on the analytical estimates in Table 5. Figure 1 presents the simulated power estimates for scenarios 1 and 2.

**TABLE 5:** Analytical sample size illustrations for 5 statistical methods

	Scenario 1		Scenario 2	
	Experimental	Control	Experimental	Control
Parameters				
Pr [died by day 90]	0.110	0.273	0.151	0.245
Pr [LT by day 90]	0.068	0.052	0.061	0.039
Pr [alive, LT-free at day 90]	0.822	0.675	0.788	0.716
λ (died by day 90)^a^	1.295×10^−3^	3.543×10^−3^	1.189×10^−3^	3.123×10^−3^
Statistical analysis	Projected sample size for 80% power			
Mortality	182		562	
Competing risks	182		562	
Principal stratum	186		615	
Win ratio	232		902	
Composite endpoint	272		1128	

^a^ λ (Died by Day 90) = −ln (1 − Pr[Died by day 90])/90. For this value of λ, if lifetimes on study follow an exponential distribution, then the day 90 mortality will equal that given under parameters.

Abbreviation: LT, liver transplantation.

## DISCUSSION﻿

Studying therapeutic agents for AH has become more challenging with the widespread adoption of LT as a treatment. Current studies are difficult to compare to those conducted before the routine use of LT.^3^^–^^5^ While LT offers a very high 90-day survival rate, it remains unavailable to most patients with this life-threatening condition. Therapeutic agents that can improve or stabilize AH may help make the decision to pursue LT in a more controlled environment, thereby improving the utilization of scarce donor organs. LT for AH was initially described as a rescue therapy for a small subset of patients who were likely to die without a transplant.^12^ If LT was reserved for patients at high risk of death, and if we had reliable tools to predict 90-day mortality, transplant-free survival could be a reasonable endpoint. However, this view is overly simplistic. The slow improvement trajectory, imprecise prognostic markers, uncertainty regarding the severity of underlying hepatic fibrosis, and concern about missing the window of opportunity for transplantation have all contributed to AH becoming the fastest-growing indication for transplantation in history. Given the prevalence of LT in specialized centers where AH studies are typically conducted, it is crucial to account for LT, even though it remains accessible to only a small minority of patients with AH. To the extent possible, we aim to ensure that the methods used in clinical trials are widely applicable. The hepatology community and regulatory agencies must reach a consensus on this issue to facilitate the development of therapeutic agents for AH. In this paper, we present multiple endpoint options, highlighting their respective strengths and weaknesses. As demonstrated in our examples, the choice of endpoint and statistical methods can significantly influence the sample sizes needed to achieve statistical significance.

Readers should keep in mind that many of these design decisions involve a trade-off between a less variable and a more representative study population. Given a set of entry criteria, sponsors of late-stage trials have 3 options to address variability. If the data can be cleanly divided into subpopulations, analyses can be stratified by those subpopulations. Alternatively, if such a division is not possible, the analysis can incorporate covariates. Lastly, sponsors can use statistical methods that account for additional data variability. One specific trade-off we recommend is to stratify subject randomization by clinical site. This approach accepts the increased risks of unintentionally unblinding subjects and the potential for small study-wide imbalances in treatment assignments in exchange for balancing site-dependent variations in patient population and standard of care across treatment groups.

Three important factors in the design of clinical trials for AH are effect size, sample size, and statistical method. The figure illustrates power analyses using data from the AHFIRM trial (Figure 1), revealing several key observations. The choice of statistical method significantly influences the study power. For example, achieving 80% power requires ~150 patients if mortality is the primary endpoint, but nearly 300 patients if the composite endpoint (death or liver transplant) is used. Effect size is also a critical factor in power calculations. A clinical trial to assess a drug with the effect size described in scenario 2 may be unrealistic.

A striking finding in the AHFIRM trial was the geographic differences in outcomes. While regional differences in patient demographics and outcomes are well-documented,^40^ we did not anticipate the magnitude of these disparities in the trial. Future studies should evaluate whether patient and management differences justify conducting separate studies in certain regions. The core of a successful trial lies in the comparability between groups. Although LT is now widely performed in US liver centers, randomization at each individual site may help reduce variability in LT and other practices. As noted, investigators should address the issue of multiple comparisons if using more than 1 statistical method and carefully assess whether geographic differences justify separate regional studies. The hierarchy presented here is based on our experience with the AHFIRM trial and is expected to remain relevant as the management of AH continues to evolve.

In conclusion, the selection of endpoints and statistical methods in clinical trials for AH is crucial in determining the feasibility and validity of study outcomes. Careful consideration of effect size, sample size, and geographic differences will be essential as the field progresses and the management of AH adapts to new therapeutic developments.
